# CRISPR-Cas Technology for Bioengineering Conventional and Non-Conventional Yeasts: Progress and New Challenges

**DOI:** 10.3390/ijms242015310

**Published:** 2023-10-18

**Authors:** Yuanyuan Xia, Yujie Li, Wei Shen, Haiquan Yang, Xianzhong Chen

**Affiliations:** 1 Key Laboratory of Industrial Biotechnology, Ministry of Education, School of Biotechnology, Jiangnan University, 1800 Lihu Avenue, Wuxi 214122, China; yyxia@jiangnan.edu.cn (Y.X.); 6190201020@stu.jiangnan.edu.cn (Y.L.); shenwei_micro@163.com (W.S.); haiquanyang@jiangnan.edu.cn (H.Y.); 2 School of Biotechnology, Jiangnan University, Wuxi 214122, China

**Keywords:** gene editing tools, CRISPR-Cas, *Saccharomyces cerevisiae*, non-conventional yeasts, yeast cell factories

## Abstract

The clustered regularly interspaced short palindromic repeats (CRISPR) and CRISPR-associated protein (CRISPR-Cas) system has undergone substantial and transformative progress. Simultaneously, a spectrum of derivative technologies has emerged, spanning both conventional and non-conventional yeast strains. Non-conventional yeasts, distinguished by their robust metabolic pathways, formidable resilience against diverse stressors, and distinctive regulatory mechanisms, have emerged as a highly promising alternative for diverse industrial applications. This comprehensive review serves to encapsulate the prevailing gene editing methodologies and their associated applications within the traditional industrial microorganism, *Saccharomyces cerevisiae*. Additionally, it delineates the current panorama of non-conventional yeast strains, accentuating their latent potential in the realm of industrial and biotechnological utilization. Within this discourse, we also contemplate the potential value these tools offer alongside the attendant challenges they pose.

## 1. Introduction

**Figure 1:**
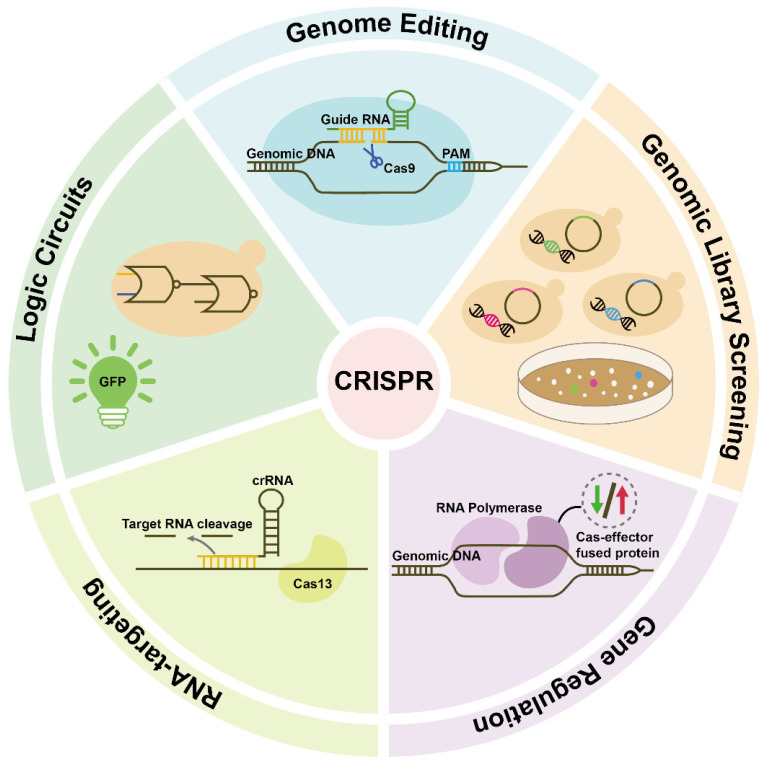
The application fields of CRISPR technology.

The development of the clustered regularly interspaced short palindromic repeats (CRISPR) and CRISPR-associated protein (CRISPR-Cas) technology has greatly accelerated the applications of molecular tools in the fields of medicine, gene therapy and industrial microbiology [1,2,3,4,5]. The groundbreaking CRISPR technology, initially unearthed within prokaryotic genomes and subsequently adapted for use in diverse cell types, has once again seized the spotlight. CRISPR is assuming a pivotal role across numerous domains of scientific research (Figure 1) [6,7,8,9,10].

Currently, CRISPR-Cas is widely applied in both eukaryotic and prokaryotic hosts, and its powerful gene editing efficiency has also facilitated the construction of microbial cell factories for the production of target compounds. Among various microbial host cells, *Saccharomyces cerevisiae* stands out due to its uncomplicated structure and post-translational modification system [11]. This single-celled eukaryote is frequently employed for the establishment of microbial cell factories. Engineered yeast has the capacity to generate anticancer and antiviral drugs within the medical field, petroleum-derived chemicals, health-oriented products in the food industry, and microbial fertilizers in agriculture [12,13], conforming to the call of green chemical technology. In contrast to prokaryotes, the advancement of yeast metabolic engineering was previously hindered by the challenges of genetic manipulation. However, with the strides made in molecular biology, high-efficiency gene editing technologies have been explored in both the conventional yeast *S. cerevisiae* and non-conventional yeasts such *as Schizosaccharomyces pombe*, *Pichia pastoris*, *Yarrowia lipolytica*, *Candida tropicalis*, *Starmerella bombicola*, *Methylotrophic yeasts*, among others. These endeavors have been especially prominent within the realm of the biotechnology industry (Table 1). 

## 2. Development of CRISPR System

### 2.1. Principle and Applications of CRISPR Cas9 System

The CRISPR-Cas tool, which recognizes specific DNA with the guidance of a simple RNA, ushered in a new era of precise gene edition [4]. The CRISPR-Cas system is an adaptive immune defense mechanism that exists in bacteria and archaea. It consists of a cluster of regularly repeated short palindromes (CRISPR) and its accessory proteins (Cas). CRISPR array transcription produces CRISPR RNA (crRNA), which binds to Cas nuclease and acts on target nucleic acid through specific base complementary pairing [4]. Cas proteins have been divided into two categories, among which the single protein with multiple domains belongs to Class 2 [30]. *Streptococcus pyogenes* Cas 9 (SpCas9) is the earliest and the most widely used representative Class 2 Cas effector. The artificially constructed single specific RNA chimera [31] makes it possible to use this system as a genome editing tool. 

In yeast cells, CRISPR-Cas9 editing tools have been explored to edit endogenous genes, adapt bacterial genomes cloned within [32], and study the relationship between phenotypes and genotypes [33]. In addition, considering the biosafety issues possibly caused by escaping yeast strains from the laboratory, researchers have also developed a CRISPR-Cas9 gene drive system to reduce the risk of unnecessary genome editing [34].

### 2.2. Selection of Nucleases

Among the common CRISPR systems, on the basis of Cas9, multiple nucleases have been utilized these days. The CRISPR-nickase system [35] utilized a Cas9 nickase to overcome the base editing limitations seen in the traditional CRISPR-Cas9 system. Operating effectively up to 53 base pairs from the nick site, this system exhibited precise cuts and no off-target editing compared to CRISPR-Cas9 in experiments. This approach empowers rapid, site-specific, and accurate base editing in yeast, enabling yeast mutant construction in just five days. 

**Figure 2:**
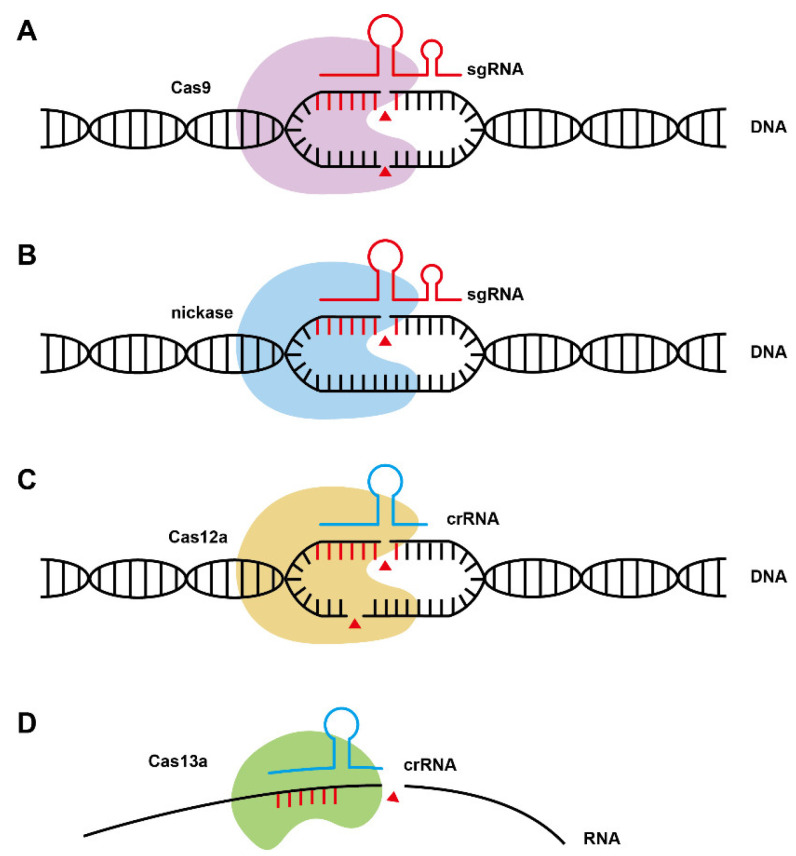
Illustration of mechanisms for different CRISPR systems. (**A**) CRISPR-Cas9 system: the first system developed and applied in the construction of microbial cell factories. Cas9 contains RuvC and HNH nuclease domains. It forms a complex with artificially designed sgRNA and binds to the PAM region of DNA, cutting the double-stranded DNA with specificity. (**B**) CRISPR-Nickase system: Nickase is obtained by inactivating the nuclease activity of one of the functional domains of Cas9 protein, which can complete the cleavage of single-stranded DNA. (**C**) CRISPR-Cas12a system: Cas12a protein is smaller in size than Cas9 protein, with only RuvC nuclease domain. It can identify multiple types of PAM, and only requires simple crRNA to target the specific DNA region. The formed DNA gap is asymmetric, forming a 5–7 nt overhang structure, which is beneficial to recombination repair. (**D**) CRISPR-Cas13a system: Cas13a can be guided by crRNA, binding to a specific site of single-stranded RNA. Compared with the previous nucleases, the definition of RNA cutting sites is not clear; red triangles represent gaps caused by nucleases.

Except for Cas9, other Cas effectors, such as Cas12a(Cpf1) and Cas13a have also been explored (Figure 2). Cpf1 [36] is a novel single RNA-guided CRISPR-Cas endonuclease with unique attributes for genome editing. In practical applications, *FnCpf1* [37] demonstrated impressive efficacy and precision, achieving DNA recombination repair with a remarkable 100% efficiency. Notably, it could simultaneously edit four yeast genes. Leveraging its RNA-targeting characteristics, Cas13a can effectively suppress gene expression levels, primarily advancing research in mammal and plant cells. Apart from optimizing the Cas protein, it is possible to elevate the efficiency of genome precision editing by co-expressing or fusing Rad52 and Brex27 with Cas9 [38]. Additionally, the transcription of sgRNA can be fine-tuned through the utilization of promoters with varying degrees of strength [39].

### 2.3. Expression Strategy of gRNA

Nuclease is the scissors in the cell, while gRNA responsible for guidance, is also important. In order to attain multi-gene editing, researchers can realize tandem expression of gRNA by using RNA polymerase III promoter, the hammerhead type (HH) ribozyme, the hepatitis D virus (HDV) ribozyme, and tRNA to promote simultaneous editing of multiple genes. Among these, U3 and U6 serve as common early RNA polymerase III promoters for gRNA production. Differently from the type II promoter responsible for mRNA, the transcription of the RNA polymerase type III promoter will not be actively transported to the cytoplasm. However, many RNA polymerase type III promoters are not yet clear in many species, and the scope of use is very limited as well [40,41].

To devise a universal gRNA expression strategy for diverse microbial cells, researchers crafted an artificial gene known as RGR. The designed RNA molecule contains HH ribozyme at the 5′ end, the gRNA located in the middle, and HDV ribozyme located at the 3′ end. The ribozyme can catalyze the cleavage of a specific site in the RNA molecule. After the predetermined site is self-cleaved, mature gRNA will be released. The artificial gene can direct DNA cleavage both in vitro and in yeast cells [42]. In addition, referring to the maturation process of tRNA in organisms, tRNA will also undergo ribozyme cleavage at specific sites. The first tRNA used in plant genome editing shows the potential of a single polycistronic gene to produce large amounts of gRNA [43]. In *S*. *cerevisiae*, by designing a gRNA-tRNA array, combined with the Golden Gate reaction strategy, six genes can be destroyed efficiently in a short time [44]. In addition to endogenous tRNAs, similarly, multiple gRNAs can be generated from a single transcript by using the recognition sequence of exogenous cleavage factors Cys4 [45].

## 3. Applications of CRISPR Systems in *S. cerevisiae*

### 3.1. Genome Editing 

In the model engineering strain, *S. cerevisiae*, CRISPR systems have been researched more deeply compared with those in other types of yeast. Using CRISPR strategies for gene editing in *S. cerevisiae* typically involves the following steps, including selecting a target gene, designing sgRNA sequences, constructing plasmids containing CRISPR elements, marker gene, and homology arms, transforming the plasmids or linearized DNA fragments along with donor DNA, and conducting selection and verification processes. From the initial research on the tolerance of Cas9 and guide RNA (gRNA) mismatches in vivo and in vitro to today’s study [46], many related genetic tools are becoming mature, such as some vector toolboxes: MoClo-based yeast chromosome modification kit [47], unmarked and commercialized EasyClone-MarkerFree [48], and a controllable copy number plasmid [49] constructed with dominant markers. Regarding the effects of CRISPR-relevant tools, researchers can knock out fragments over 30 kb in *S. cerevisiae* [50]. By using the delta site as a targeting site, the Di-CRISPR platform can integrate up to 18 copies of the gene in one step [51]. In addition, the CRISPR Cas9 system can construct auxotrophic mutants of industrial tetraploid yeast strains [52]. In the research of *S. cerevisiae* genomics, mCRISPR combined CRISPR-Cas9 and transformation-associated recombination (TAR) to enable single-marker multiplexed promoter engineering of large gene clusters, which enabled efficient multiplexed engineering of natural product biosynthetic gene clusters [53].

### 3.2. Genome Library Screening

Efficient screening of ideal industrial yeast strains depends on the establishment of related libraries. Taking advantage of Cas9 mutants with confirmed genetic variations (deletion, substitution, and insertion) in yeast, a genome-wide library established in *S. cerevisiae*-short, trackable, integrated cellular barcodes (MAGESTIC) achieved saturation editing of the essential gene SEC14, identifying amino acids highly related to inhibiting lipid signaling [54]. Guo and colleagues integrated next-generation sequencing technology (NGS) to construct a genome library targeting 315 small open reading frames with unclear characteristics in the yeast genome, which evaluated genes crucial for growth in various environments [55]. *S. cerevisiae* is an industrial strain commonly used for the production of ethanol. Liu and colleagues have constructed a Global Regulatory Network (MINR) with 43,020 specific mutations of 25 regulatory genes and identified yeast strains with improved ethanol tolerance [16].

### 3.3. Transcription Regulation

Common transcription regulation systems in eukaryotic cells can be divided into three generations [56], all of which use Cas proteins lacking endonuclease activity. For example, dCas9 can be obtained by mutating specific amino acids in the RuvC and HNH functional domains [57]. Similarly, other types of Cas proteins can also be utilized for transcriptional regulation [56]. The principle of the first-generation transcription control system is that when Cas protein is co-expressed with sgRNA, a DNA recognition complex is produced, and then it becomes a roadblock in the transcription process. Furthermore, fusion expression of transcription activator or transcription repressor with dCas9 can achieve gene activation or inhibition. Differently, compared with the additional modification of dCas9 in the first generation, the second generation used a special scaffold RNA to recruit specific effectors. The formed RNA aptamer–RBP pair could regulate the transcription of target genes [58]. The third-generation system is a dimerization system controlled by chemistry and light, further realizing the temporal and spatial control of gene functions.

In recent years, dCas9 has been widely applied in the regulation of the yeast metabolic network. In the glycerol synthesis pathway, by changing the target position of sgRNA, dCas9 can be used to express genes in stages (activation or inhibition) and test systematically enzyme perturbation sensitivity (STEPS) [59], in order to identify the rate-limiting steps in the metabolic pathway. dCas9 can not only fulfill individual inhibition or activation, but also achieved orthogonal transcriptional activation, transcriptional interference, and gene deletion (CRISPRAID). The three-function system containing Cas9 proteins from different sources could modularly, parallelly regulate and interfere with the metabolic network in high-throughput ways. After optimizing different combinations of inactivated nucleases, activators and inhibitors, the production of β-carotene could be increased by three times [17]. A more powerful synthetic biology tool, multi-functional genome-wide CRISPR system (MAGIC), can precisely control the defined genes in the genome. It has great application prospects in the genome-scale engineering of advanced eukaryotes [60]. 

Beyond the spatial level, synthetic biological elements can realize real-time gene regulation. Researchers have constructed and compared two dCas9-mediated systems, constitutive and inducible, with different gRNA design strategies. In this way, high production of isoprenoids was achieved and production of triglycerides was greatly improved [18].

### 3.4. Base Editing

As dCas9 and Cas9 nickase (D10A) will not cause DNA double-strand breaks, fusion expression of their functional domains with adenine/cytidine deaminase can change the corresponding DNA bases and achieve single-base editing [61]. Earlier, through the fusion of CRISPR/Cas9 lacking nuclease activity and activation-induced cytidine deaminase (AID) orthologs, the artificial synthetic complex (Target-AID) [62] can work efficiently in yeast, while in mammalian cells, Uracil DNA glycosidase inhibitors (UGI) are needed to inhibit insertions and deletions.

In addition to the developed adenine base editor ABEs [63] without causing double-strand DNA breaks, Liu and colleagues have recently built a single-base editing system [64,65], which brought a milestone breakthrough in the field of gene editing. The simplest system only contained engineered reverse transcriptase, Cas9 nickase and the guide RNA. It directly wrote new genetic information into the designated position of DNA in eukaryotic cells. Since many human diseases are related to single-base mutations, the CRISPR system has been demonstrated the potential to correct known pathogenic human genetic variants in the medical field.

### 3.5. Logic Circuit Control

By means of connecting genetic components, a genetic circuit with complex logic functions can be constructed. In order to solve the problem of the signal degradation when layered in dCas9 system, the NOR gate developed in *S. cerevisiae* based on dCas9-Mix1 has been established [66]. In a single-gene NOR logic gate, the input and output signals of the gate are programmable signal molecules-gRNAs of the same molecular type. The input end is composed of RNA Pol II pGRR promoter, and the output end is the gRNA transcript flanking with self-splicing ribozyme (RGR). On the basis of this, the NOR gate is integrated into a single yeast genome, and the fluorescent protein intensity at the output end was the signal comparing the effects of different logic circuits. It was found that digital logic circuits with up to seven gRNAs, five NOR gates, and up to seven ladders could be constructed. 

In addition, in *S. cerevisiae*, an AND logic gate was developed with a transcription activation system composed of MCP-VP64, scRNA, and dCas9 [67]. The AND logic gate consisted of two switches, and activating two independent switches made the reporter gene express. The endogenous Gal10 regulated by galactose and the heterologous transcription factor LexA-ER-AD regulated by β-estradiol controlled the constitutively expressed RNA scaffold. It is worth mentioning that the system utilized ribosome skipping T2A sequence to bind target gene and fluorescent reporter gene. This peptide could make a transcript translate multiple proteins due to ribosome jumping, so as to adapt to any target gene and ensure the reporting function simultaneously.

## 4. Applications of CRISPR System in Non-Conventional Yeast

### 4.1. Gene Editing Tools of Non-Conventional Yeasts

Differing from *S. cerevisiae*, an increasing number of non-conventional yeasts have garnered significant attention for their role in the production of high-value chemicals, oils, and recombinant proteins, for instance, *Schizosaccharomyces pombe*, renowned for its high ethanol production capability, *Methylotrophic yeasts*, which can utilize methanol as their sole carbon source and energy supply, *Yarrowia lipolytica*, recognized for its efficient conversion of carbon sources into cellular lipids, *Candida tropicalis*, a proficient producer of long-chain dicarboxylic acids, and *Starmerella bombicola*, known for its high yields of sophorolipids. Despite their numerous advantageous characteristics, engineering non-conventional yeasts remains challenging due to a significant deficit in genetic editing tools when compared to the model yeast *S. cerevisiae* [68]. Unlike *S. cerevisiae*, which can incorporate marker gene into free plasmids to express CRISPR elements, many non-conventional yeasts require the development of a screening system for gene editing. The split-marker system involves separating the components of Cas9 and sgRNA and loading them into two different segments, and the strategy facilitates the in vivo reconstruction of the selectable cassette to express CRISPR elements [69,70]. The principal advantages of this technique encompass the attainment of markerless deletions in a singular transformation, the obviation of a cloning step, and the potential for generating reconstituted strains. In contrast, the principle of the whole-marker approach entails incorporating all editing components into a single plasmid, allowing for a one-time introduction into the cell for editing [71,72]. During the process of cleaving and editing the target gene, it is customary to introduce plasmids containing CRISPR elements or linearized fragments into the host cells. In *S. cerevisiae*, both intact plasmid and TAR can be used for gene editing. TAR is the utilization of the homologous recombination system in the yeast to enable DNA fragments to recombine into a complete gene sequence within the yeast cell [73]. For non-conventional yeasts that lack free plasmids, the TAR strategy is typically employed for gene editing, whereas transformation shuttle plasmids often exhibit lower gene editing efficiency [72,74].

### 4.2. Schizosaccharomyces pombe

*Schizosaccharomyces pombe* is also a traditional model strain and the preferred model organism for chromosomal biology research. In 2014, the CRISPR-Cas9 system was developed in *S. pombe*, and the *rrk1* gene was used to construct an sgRNA expression system [75]. In 2016, the first positive selection marker (*Fex1p* and *Fex2p*) [76] increased the throughput of engineering strains constructed by the CRSIPR-Cas9 system. In 2018, a cloning-free method was developed. The gap repair in fission yeast cells can be used to assemble two linear DNA fragments. This method can be used to achieve sequence deletion, rapid point mutation knock-in, and endogenous N-terminal labeling [77]. In addition, the CRISPR-Cas9 toolbox can quickly introduce the designed auxotroph. The total number of available markers reaches four, including *leu1-Δ0, his3-Δ0, lys9-Δ0*., and *ura4-D18* [78]. In terms of network tools, the PCR-based network tool CRISPR4P [79] supported primer design for the entire process of seamless deletion of sgRNA and DNA. 

Compared with Cas9, Cas12a can identify T-enriched PAM regions and has the advantage of multi-gene editing. In 2021, the first CRISPR-Cas12a toolkit was developed in *S. pombe*, and the rrk1 promoter was adopted. The more efficient exogenous RNA pol II promoter expressed crRNA, which could edit three loci in a one-time transformation. It proved the potential of this system for efficient and rapid genome gene editing and regulation in fission yeast [80]. In *S. pombe*, CRISPR-Cas9 system has already been used in metabolic engineering [21]. By knocking out the genes encoding pyruvate decarboxylase (PDC), alcohol dehydrogenase (ADH), and glycerol-3-phosphate dehydrogenase (GPD), and introducing two copies of the D-lactate dehydrogenase (D-LDH) encoding gene, the formation of by-products was eliminated and the strain intaking glucose and cellobiose to produce D-LA was obtained. Jing and colleagues constructed the Cas13a system in *S. pombe*, tethering dCas13a to the catalytic domain of human adenosine deaminase, to restore the transposition of retrotransposon Tf1 mutants in fission yeast [75].

### 4.3. Methylotrophic yeasts

Methylotrophic yeasts like *Komagataella phaffii* (formerly known as *Pichia pastoris*) and *Ogataea polymorpha* find extensive use in both fundamental research and biotechnological applications, often in contexts necessitating genetic alterations [81,82]. *Pichia pastoris* is often used to produce heterologous proteins, in which CRISPR-Cas9 system using RNA pol II promoter was first established in 2016 [83]. In contrast to *S. cerevisiae*, *K. phaffii* predominantly relies on non-homologous end joining (NHEJ). The efficiency of non-homologous recombination of NHEJ is nearly 100%, while the efficiency of specific integration of the homologous donor cassette by homologous recombination (HR) was only 20%. As *Ku70p* gene is related to the NHEJ repair pathway, with the Ku70p gene knocked out, the efficiency of HR specific integration was greatly improved [84]. Recently, the CRISPR–Cas9 system has been established and fine-tuned in *K. phaffii* through the assessment of various codon-optimized Cas9 genes, sgRNAs, and promoters for the expression of Cas9 and sgRNA [83]. In *K. phaffii*, a CRISPR/Cas-based toolbox has also been developed [85]. It was found that panARS from *Kluyveromyces lactis* increased the editing efficiency by nearly 10 times, compared to the commonly used endogenous ARS in a previous report [86]. And sgRNA expression through the RNA pol III promoter (SER promoter, tRNA) was proved successful [87]. In addition, the CRISPR-Cas9 system containing free sgRNA plasmids and CRISPRi system has also been developed in *K. phaffii* [88].

In regard to the applications of the CRISPR-Cas9 system, Hou and colleagues [89] edited the transcription activator Mxr1 on the genome, and precisely deleted or inserted specific bases at key amino acid positions. Liu [23] developed a one-step integration method in the non-homologous-end-joining defective strain Δku70, tested the efficiency of double-gene and triple-gene co-integration, and successfully assembled biosynthetic pathways of 6-methylsalicylic acid and 3-methylcatechol via one-step integration. In *K. phaffii*, for producing isopentanol, Siripong [22] knocked out the endogenous valine and leucine synthesis pathway related genes, and constructed heterologous keto acid degradation pathway. The engineering *K. phaffii* strain produced isopropanol with a titer of 191.0 mg/L, which was the highest yield in unconventional yeast reported.

### 4.4. Yarrowia lipolytica

*Yarrowia lipolytica* is a yeast strain with low pH tolerance, able to utilize various renewable raw materials such as pentose, glycerin, and volatile fatty acids. It has been applied in producing lipids, oleochemicals, and fuels. However, metabolic engineering in this yeast faces challenges due to limited HR efficiency and a lack of adequate genetic tools. In 2016, a double-plasmid-based CRISPR system with sgRNA expressed by the RNA pol III promoter was developed [90]. And the combination of the RNA pol II promoter and ribozyme can also be applied to *Y. lipolytica*. Based on the CRISPR system, a variety of efficient molecular genetic tools have been subsequently developed, expected to accelerate the construction of complex gene circuits in lipid yeast, such as the PCASyl system, YailBricks vector, and the EasyCloneYALI gene toolbox. The PCASyl [91] system was based on a single plasmid and could perform multiple rounds of gene editing in different *Y. lipolytica* strains. The engineered vector YailBricks is suitable for *Y. lipolytica* modules and combinatorial pathway engineering [92]. The EasyCloneYALI gene toolbox [93] allowed gene expression vectors to integrate specific genomic sites without markers and delete genes without markers as well. In previous studies, there has been a recovery time of 2–4 days after first turn of editing. The toolbox used non-replicating DNA repair templates to achieve efficient genome editing. Consequently, the single gene knockout efficiency reached 90%, and the double gene knockout efficiency was 6–66%, and related vectors have been commercialized. In addition, the CRISPR-Cas12a gene editing system [94] was recently developed in *Y. lipolytica*. What is different from the editing effect of Cas9 is that after non-homologous recombination repair, the PAM site can be retained, allowing secondary editing of the same target gene so as to improve editing efficiency.

In *Y. lipolytica*, gene editing systems have been used to identify key genes in metabolic pathways and important mutations in genomic libraries. In order to clarify the xylose metabolism pathway [95], XKS or XDH genes in the genome were destroyed by introducing frameshift mutations. In the process of whole-genome screening of crucial strains, it is inevitable to solve the difficulty of identifying functional sgRNA. Considering the difference between *S. cerevisiae* and *Y. lipolytica*, the DNA repair of the latter mainly relies on NHEJ. In the absence of NHEJ and homologous repair templates in haploid strains, Cas9-induced double-strand breaks are likely to cause cell death, and therefore, the editing efficiency can be evaluated based on cell viability. In *Y. lipolytica*, the CRISPR-dCas9 activation system activated the transcription of two different natural β-glucosidase genes, improving the growth of the strains relying on cellobiose as the sole carbon source [96].

### 4.5. Candida tropicalis

*Candida tropicalis* is an industrial diploid yeast commonly used to produce long-chain dibasic acids. In the developed CRISPR-Cas9 system, RNA pol II [27] promoter and ribozyme or a combination of heterologous tRNA [97] and ribozyme can be used to express sgRNA. Zhang tested the effect of expressing an integrated CRISPR–Cas9 cassette or a transient CRISPR–Cas9 cassette, which demonstrated that the CRISPR-Cas9 system could promote the in vivo assembly of multiple DNA fragments and stably integrate the target locus. Furthermore, the synthesis pathway of β-carotene and its derivatives in *C. tropicalis* was established with the help of the system [27].

### 4.6. Starmerella bombicola

*Starmerella bombicola* is known to produce sophorolipids which are carbohydrate-based, amphiphilic biosurfactants [98,99]. Due to the initial lack of effective gene editing tools, the screening of high-yield sophorolipid-producing strains relied on directed evolution using physical mutation, chemical mutation, and compound mutagenesis methods [100,101]. Shi et al. [28] were the first to establish the CRISPR-Cas9 gene editing tool in *S. bombicola*, enabling single-gene, double-gene, and triple-gene knockouts. Targeted knockouts of the single gene PXA1 and the double gene exhibited efficiencies of up to 100%, while the triple-gene knockout achieved an efficiency of 16.5%. Through a CRISPR-Cas9-integrated expression system, an in vivo one-step assembly was used to enhance the expression levels of two key genes via promoter engineering. An acidic sophorolipid-producing strain with a yield of 99.5 g/L in a 5 L fermentor was constructed using CRISPR-Cas9 system [28]. Subsequently, Zhang et al. established the CRISPR-Cas12a gene editing tool in *S. bombicola*. This tool was further expanded to simultaneously edit two genes (gene editing efficiency of 18%) and three genes (gene editing efficiency of 13.8%). Using this system, they successfully biosynthesized polyhydroxyalkanoates (PHA) from scratch in *S. bombicola*, with PHA content and DCW reaching 15.2% and 28.2 g/L, respectively [29].

### 4.7. Other Yeasts

Apart from the aforementioned yeasts, improvements in editing efficiency have also been achieved through the targeted design of gRNAs in *Candida albicans* [102], *Scheffersomyces stipites* [103], and *Ogataea polymorpha* [104]. Furthermore, the utilization of artificially fused promoters demonstrated enhanced functionality within these yeast species.

## 5. Conclusions

From ZFNs and TALEN systems to the rapidly advancing CRISPR system, the current landscape of gene editing has become notably more convenient, versatile, and efficient. The utilization of the CRISPR system in conventional yeasts has reached a relatively mature stage, and its related technologies can also be extrapolated to non-conventional yeasts. This acceleration in technology has hastened the creation of diverse yeast cell factories. Within yeast cells, the novel gene editing technology has significantly truncated the research timeline for scientists. In diploid yeast cells, the traditional recombination method necessitated nearly two weeks to complete, whereas the current CRISPR-based approach yields experimental results within just one week [27]. Furthermore, the innovation of CRISPR editing tools has enabled the streamlined reconstruction of metabolic pathways through one-step integration [23], offering a pivotal impetus for advancements in metabolic engineering. In conjunction with NGS and bioinformatics, the mutant library constructed via CRISPR systems is becoming more systematically designed, and data visualization is accessible to both researchers and readers, facilitating a deeper understanding of the outcomes.

There remain several challenges that scientists must confront. Firstly, the off-target effects associated with various gene editing tools can lead to unintended edits. In-depth research into the characteristics of commonly used Cas effectors will facilitate the broader application of diverse CRISPR systems, ensuring that these nucleases can be harnessed effectively to suit specific requirements. Secondly, compared with the conventional yeast, both in terms of quantity and functionality, the development of efficient gene editing tools is an urgent necessity. These tools should aim to replace the time-consuming conventional molecular biological techniques. Thirdly, the newly devised prime editing tools hold promising potential in the medical field and could be adapted for precise base editing in microorganisms. Lastly, given the often-competitive nature between growth and production phases, temporal and logical regulation has emerged as a focal point in metabolic engineering. The design of logical circuits and dynamic control elements based on CRISPR systems requires further exploration to find applications in yeast fermentation.

## Figures and Tables

**Table 1:** CRISPR-related applications in yeast metabolic engineering.

Strain	Tool	Strategy	Production	Result	Reference
*S. cerevisiae*	CRISPR-Cas9: genome editing	Iteratively integrate the genetic parts into the repetitive genomic sequences using robotic automation	Cellulase, isobutanol	Optimize cellulase expression, isobutanol production, glycerol utilization, and acetic acid tolerance on a genome scale	[14]
	CRISPR-Cas9: genome editing	Generate a yeast cell factory with a simplified lipid metabolism network	Free fatty acids	Increase free fatty acid up to 40-fold and suggest the importance of phospholipid hydrolysis in free fatty acid production	[15]
	CRISPR-Cas9: genome library screening	MINR library construction	Ethanol	Obtain mutants with increased ethanol and/or glucose tolerance	[16]
	CRISPR-Cas9/dcas9: genome editing, transcription regulation	Crispraid ^a^	β-carotene	Increase the production of β-carotene 3-fold	[17]
	CRISPR-dcas9: transcription regulation	Construct inducible system and optimize grna design	Isoprenoid, triacylglycerols	Enable significant changes in isoprenoid production triacylglycerols	[18]
	CRISPR-Cas9: genome editing, transcription regulation	Crisprare ^b^	α-santalene	Improve the production of α-santalene 2.66-fold in a single step	[19]
	CRISPR-Cas9/dcas9: genome editing, transcription regulation	Construct a system, SWITCH, to iteratively alternate the strains between a genetic engineering state and a pathway control state.	Naringenin	Exploit the genetic engineering state to insert the genes necessary for naringenin production and downregulate an essential byproduct gene TSC13 in thepathway control state	[20]
*S. pombe*	CRISPR-Cas9: genome editing	Delete and integrate the target genes	D-lactic acid	Firstly, use a CRISPR-Cas9 system to construct a metabolically engineered *S. pombe* strain	[21]
*P. pastoris*	CRISPR-Cas9: genome editing	Delete PDC1 gene	Isopentanol	Reach the highest titer reported to date in a nonconventional yeast	[22]
	CRISPR-Cas9: genome editing	Develop a multi-loci gene integration method	6-methylsalicylic acid and 3-methylcatechol	Assemble the biosynthetic pathways of 6-methylsalicylic acid and 3-methylcatechol with one step integration method	[23]
*Y. lipolytica*	CRISPR-Cas9: genome editing	Construct efficient β-carotene-producing *Y. Lipolytica* cell factories	β-carotene	Obtain 4.5 g/L β-carotene through a combination of genetic engineering and culture optimization	[24]
	CRISPR-Cas9: genome editing	Analyze the secretion of heterologous proteins in *Y. lipolytica* PO1f.	β-glucosidase	Indicate that *Y. lipolytica* is a promising host for the secretion of heterologous high-molecular-weight proteins (>100 kda)	[25]
	CRISPR-Cas9: genome library screening	Quantify sgrna function utilizing high throughput	/	Identify novel mutations for metabolic engineering of high lipid accumulation	[26]
*C. tropicalis*	CRISPR-Cas9: genome editing	One-step multi-gene pathway assembly	Β-carotene	Present a platform for the biosynthesis of β-carotene and its derivatives	[27]
*S. bombicola*	CRISPR-Cas9	A CRISPR-Cas9 system-mediated genetic disruption and multi-fragment assembly	Acidic sophorolipids	Engineered a strain for the biosynthesis of acidic sophorolipids through the in vivo assembly of multiple DNA fragments (10 kb)	[28]
	CRISPR-Cas12a	A CRISPR-Cas12a system for multi-gene editing (CCMGE)	Polyhydroxyalkanoate	Engineered a strain for the biosynthesis of polyhydroxyalkanoate using CCMGE	[29]

^a^ CRISPR system combining transcriptional activation, transcriptional interference, and gene deletion. ^b^ CRISPR system enabling simultaneous gene activation, repression, and editing with a single Cas9-VPR protein.

## Data Availability

Not applicable.
